# SARS-CoV-2 Vaccination Coverage and Key Public Health Indicators May Explain Disparities in COVID-19 Country-Specific Case Fatality Rate Within European Economic Area

**DOI:** 10.7759/cureus.22989

**Published:** 2022-03-09

**Authors:** Vasileios P Papadopoulos, Anatoli Emmanouilidou, Marios Yerou, Stefanos Panagaris, Chousein Souleiman, Despoina Varela, Peny Avramidou, Evangelia Melissopoulou, Chrysostomos Pappas, Zoi Iliadou, Ilias Piperopoulos, Vasileios Somadis, Anestis Partsalidis, Eleni Metaxa, Ioannis Feresiadis, Dimitrios Filippou

**Affiliations:** 1 Department of Internal Medicine, General Hospital of Xanthi, Xanthi, GRC; 2 Surgery, National and Kapodistrian University of Athens, Athens, GRC

**Keywords:** smoking, health spending, vaccination, european economic area, case fatality rate, covid-19

## Abstract

Aim

To investigate the reasons for disparity regarding the country-specific COVID-19-related case fatality rate (CFR) within the 30 countries of the European Economic Area (EEA).

Materials and methods

Data regarding population, area, COVID-19-associated infections/deaths, vaccination, life expectancy, elderly population, infant mortality, gender disparity, urbanization, gross domestic product (GDP), income per capita, health spending per capita, physicians, nursing personnel, hospital beds, ICU beds, hypertension, diabetes, obesity, and smoking from all EEA countries were collected from official sources on January 16, 2022. Correlation coefficients were computed, and optimal scaling using ridge regression was used to reach the most parsimonious multivariate model assessing any potential independent correlation of public health parameters with COVID-19 CFR.

Results

COVID-19 CFR ranges from 0.1% (Iceland) to 4.0% (Bulgaria). All parameters but population density, GDP, total health spending (% of GDP), ICU beds, diabetes, and obesity were correlated with COVID-19 CFR. In the most parsimonious multivariate model, elderly population rate (P = 0.018), males/total ratio (P = 0.013), nurses/hospital beds (P = 0.001), physicians/hospital beds (P = 0.026), public health spending (P = 0.013), smoking rate (P = 0.013), and unvaccinated population rate (P = 0.00005) were demonstrated to present independent correlation with COVID-19 CFR. In detail, the COVID-19 CFR is estimated to increase by 1.24 times in countries with vaccination rate of <0.34, 1.11 times in countries with an elderly population rate of ≥0.20, 1.14 times in countries with male ratio values ≥0.493, 1.12 times in countries spending <2,000$ annually per capita for public health, 1.14 and 1.10 times in countries with <2.30 nurses and <0.88 physicians per hospital bed, respectively, and 1.12 in countries with smoking ratio ≥0.22, while holding all other independent variables of the model constant.

Conclusion

COVID-19 CFR varies substantially among EEA countries and is independently linked with low vaccination rates, increased elderly population rate, diminished public health spending per capita, insufficient physicians and nursing personnel per hospital bed, and prevalent smoking habits. Therefore, public health authorities are awaited to consider these parameters in prioritizing actions to manage the SARS-CoV-2 pandemic.

## Introduction

COVID-19 case fatality rate (CFR), described as the ratio of COVID-19 associated deaths/confirmed SARS-CoV-2 infections, is a measure assessing the lethality of COVID-19 disease; CFR varies impressively within the 30 countries of the European Economic Area (EEA) [1]. Nevertheless, the imbalance in country-specific COVID-19 CFR is a worldwide phenomenon [2].

CFR does not account for the time from disease onset to death or total population; despite that, CFR is still difficult to be measured with accuracy. The main reason is that the confirmed SARS-CoV-2 infections are always less than actual cases, while COVID-19-associated deaths are far closer to the accurate number. Therefore, the current CFR is possibly overestimated. In fact, the more tests performed, the more SARS-CoV-2 infections are confirmed, minimizing the hazard of CFR overestimation [3]. In addition, CFR is often confused with the overall mortality rate (OMR), measured as the ratio of COVID-19-associated deaths/population/year. In fact, OMR is rather a measure of the individual risk of dying from the disease rather than reflecting the lethality of the disease [3]. Major risk factors for COVID-19 mortality were recognized. Age has been documented as a significant determinant of surviving from COVID-19; therefore, it would be reasonable to assume that the older a population gets, the more vulnerable it becomes [4]. In addition, the male sex has been reported to be at an increased risk of dying from COVID-19 [5]. Furthermore, comorbidities such as hypertension, diabetes, obesity, and smoking have been described as key contributors to an undesirable outcome [6]. Other parameters, such as medical [7], genetic [8], environmental [9], physical [10,11], mental/psychological [12], social [13], economical [14], and political [15], whose effect cannot be precisely determined, at least at an individual level, are believed to contribute to country-specific CFR disparities. Last, concerning vaccination for SARS-CoV-2, it has been reported that vaccination uptake is closely associated with elderly people saved, independently of the vaccine type administered [16]. Emerging data further support the efficacy and effectiveness of COVID-19 vaccines in terms of public health consequences [17].

Public health authorities rely on rigid data and optimal models to prioritize strategies for measures against COVID-19 spread, such as vaccination [18]. To contribute to these efforts, the present work aims to investigate the factors that potentially influence differences in country-specific CFR within EEA. This is done using data obtained from official sources to conclude the most parsimonious multivariate model assessing any potential independent correlation of SARS-CoV-2 vaccination coverage and public health parameters with COVID-19 CFR.

## Materials and methods

Data regarding public health indicators from all 30 countries of the EEA were collected from official sources such as European Centre for Disease Prevention and Control (ECDC), Eurostat, WHO, Organization for Economic Co-operation and Development (OECD), World Bank Group, and Institut national d'études démographiques (INED). The timeframe of the study was from January 24, 2020, when the first documented case of SARS-CoV-2 infection was detected in Europe, to January 16, 2022, when all sources were accessed simultaneously [19]. A public health indicator was considered eligible if 1) it refers to either population demographics, or general health status, or general financial indices, or determinants of health and risk factors specially focused on COVID-19, or public health interventions, or public health services, or public health capacity; 2) it is population-based at the national level so as to allow comparisons between countries; 3) it can be retrieved from routinely available data; and 4) it derives from sources that ensure validity and reliability [20,21]. In detail, the following parameters were considered: Population [22], area [23], confirmed SARS-CoV-2 infections [1], COVID-19-associated deaths [1], infected population ratio (IFR, computed as the ratio of confirmed SARS-CoV-2 infections/population), CFR of COVID-19 (computed as the ratio of COVID-19-associated deaths/confirmed SARS-CoV-2 infections), total population vaccination rate for SARS-CoV-2 [24], life expectancy (years) [25], elderly population (>65 years; rate) [26,27], infant mortality (per 1,000 live births) [28-30], gender disparity (males rate) [31,32], population density (residents per km2) [33], urbanization (urban population rate) [34], gross domestic product (GDP; millions $) [35,36], income per capita ($) [37,38], health spending (% of GDP) [39,40], public health spending per capita ($) [41-43], private health spending per capita ($) [41-43], total health spending per capita ($) [41-43], physicians (per 1,000 residents) [44], nursing personnel (per 1,000 residents) [45-47], hospital beds (per 1,000 residents) [48-50], ICU beds (per 100,000 residents) [51], hypertension (rate) [52-53], diabetes (rate) [54-55], obesity (BMI >30 kg/m2; rate) [56,57], and smoking (rate) [58,59].

Statistics

Correlations between public health parameters (continuous variables) were approached by Spearman’s correlation coefficient ρ (rho) in case of either outlier detection or normality violation in either Kolmogorov-Smirnov or Shapiro-Wilk tests; else, Pearson’s correlation coefficient was alternatively preferred. Benjamini-Hochberg correction was applied when multiple hypotheses were tested simultaneously. Optimal scaling using ridge regression and 10x cross-validation was performed to assess any potential independent correlation of public health parameters (incorporated as independent ones after discretization into a maximum of seven categories) with CFR of COVID-19 (handled as continuous, dependent variable). All independent variables that presented either as P > 0.20 or tolerance ≤0.20 were not considered during the process towards the detection of the most parsimonious model. The level of statistical significance was set to P = 0.05. SPSS® Statistics 26.0.0.0 (IBM, Armonk, NY, USA) was used for statistical analysis. GraphPad Prism 9.3.1 was used to produce scattergrams and heat maps for visualization purposes. Concerning scattergrams, the ±95% confidence bands of the best-fit-line are depicted only in the case of statistically significant correlations.

## Results

Descriptive statistics

As derived from sources referred to in the “Materials and Methods” section, all raw data are included in Table 1.

**Table 1:** Detailed data from EEA countries used in the study. EEA: European Economic Area; GDP: Gross domestic product; OMR: Overall mortality rate; CFR: Case fatality rate.

Country	SARS-CoV-2 Infections	COVID-19 Deaths	COVID-19 CFR	COVID-19 OMR	Unvaccinated Population Rate for SARS-CoV-2	Life Expectancy	Elderly Population Rate	Infant Mortality Per 1,000	Males Ratio	Population Density (Per km^2^)	Urbanization Rate	GDP (Million $)	Income Per Capita ($)	Health Spending as % of GDP	Public Health Spending Per Capita ($)	Private Health Spending Per Capita ($)	Physicians/Hospital Beds	Nurses/Hospital Beds	ICU Beds	Hypertension Rate	Diabetes Rate	Obesity Rate	Smoking Rate
Austria	1392678	13450	0.0097	0.000761	0.26	81.3	0.190	3.4	0.493	107.6	0.59	430.95	35390	10.43	3892.83	1433.60	0.74	0.99	5.31	0.22	0.066	0.17	0.25
Belgium	2379053	28589	0.0120	0.001250	0.24	80.9	0.191	3.7	0.496	377.3	0.98	515.33	33880	10.66	3723.07	1189.63	0.57	2.13	4.97	0.17	0.048	0.16	0.21
Bulgaria	806977	31922	0.0396	0.002333	0.72	73.6	0.216	5.6	0.486	63.4	0.76	69.11	6830	7.13	397.64	292.27	0.57	0.64	5.95	0.27	0.067	0.13	0.38
Croatia	794190	13006	0.0164	0.001629	0.46	77.8	0.210	4.0	0.482	72.8	0.58	55.97	11720	6.98	843.93	170.25	0.64	1.12	3.99	0.37	0.052	0.23	0.36
Cyprus	218374	672	0.0031	0.000379	0.31	82.3	0.163	2.6	0.500	95.7	0.67	23.80	23770	7.01	836.52	1110.54	1.24	1.18	3.41	0.19	0.093	0.15	0.28
Czechia	2573945	36799	0.0143	0.001738	0.37	78.3	0.199	2.6	0.492	138.2	0.74	245.35	17340	7.83	1460.21	305.58	0.61	1.27	4.04	0.26	0.053	0.19	0.30
Denmark	1056389	3453	0.0033	0.000299	0.20	81.6	0.199	3.0	0.497	138.5	0.88	356.08	48150	9.96	5214.66	1002.11	1.62	4.12	2.47	0.19	0.056	0.16	0.16
Estonia	262482	1967	0.0075	0.000748	0.38	78.6	0.200	1.6	0.474	30.5	0.69	30.65	15010	6.73	1142.58	409.99	0.76	1.46	3.32	0.23	0.070	0.21	0.18
Finland	361553	1700	0.0047	0.000155	0.25	82.2	0.223	2.1	0.493	18.2	0.86	269.75	36050	9.15	3547.37	968.12	1.39	4.45	2.61	0.27	0.058	0.20	0.15
France	13240304	126530	0.0096	0.000948	0.25	82.3	0.204	3.8	0.484	106.1	0.81	2630.32	30610	11.06	3441.17	1248.91	0.54	1.97	3.00	0.17	0.054	0.14	0.28
Germany	7835451	115337	0.0147	0.000701	0.28	81.1	0.218	3.2	0.494	235.2	0.77	3846.41	35220	11.70	4251.03	1221.16	0.56	1.71	5.95	0.26	0.053	0.19	0.24
Greece	1612869	21732	0.0135	0.001028	0.31	81.2	0.223	3.7	0.491	82.4	0.80	189.41	16170	7.84	813.74	751.00	1.49	0.89	3.64	0.20	0.051	0.16	0.42
Hungary	1327014	40237	0.0303	0.002090	0.37	75.7	0.199	3.6	0.476	107.1	0.72	155.01	12680	6.35	747.53	334.27	0.52	0.79	4.21	0.32	0.06	0.24	0.28
Iceland	43768	43	0.0010	0.000059	0.23	83.1	0.144	1.1	0.502	3.6	0.94	21.71	36030	8.57	5379.65	1151.29	1.34	6.04	2.33	0.25	0.032	0.22	0.14
Ireland	1061116	6035	0.0057	0.000609	0.23	82.8	0.144	2.8	0.496	71.9	0.64	425.89	62980	6.68	4055.94	1433.13	1.19	4.51	2.69	0.12	0.052	0.26	0.18
Italy	8155645	140188	0.0172	0.001196	0.25	82.4	0.232	2.4	0.487	201.5	0.71	1886.45	24900	8.67	2208.52	780.48	1.27	1.87	2.60	0.20	0.051	0.11	0.23
Latvia	295961	4700	0.0159	0.001255	0.32	75.7	0.205	3.4	0.461	30.2	0.68	33.51	12150	6.58	657.77	441.91	0.61	0.85	3.09	0.32	0.078	0.22	0.32
Liechtenstein	6915	72	0.0104	0.000932	0.33	81.9	0.185	0.0	0.495	244.1	0.14	6.68	175814	5.57	3081.70	6788.97	2.47	1.06	1.45	0.30	0.047	0.11	0.25
Lithuania	559980	7618	0.0136	0.001378	0.31	75.1	0.199	3.3	0.463	44.6	0.68	55.89	14030	7.01	822.83	422.52	0.72	1.48	5.20	0.30	0.078	0.18	0.28
Luxembourg	121237	931	0.0077	0.000742	0.32	81.8	0.145	4.7	0.506	239.8	0.91	73.26	82250	5.37	5288.11	851.03	0.72	2.9	3.25	0.16	0.045	0.16	0.23
Malta	62387	500	0.0080	0.000490	0.15	82.6	0.185	6.7	0.501	1595.1	0.95	14.65	20410	8.95	1747.87	1005.19	0.88	2.12	3.34	0.18	0.066	0.28	0.20
Netherlands	3465463	21127	0.0061	0.000611	0.28	81.5	0.195	3.6	0.498	507.3	0.92	913.87	40160	10.17	3444.81	1860.37	1.20	3.73	2.62	0.16	0.053	0.14	0.12
Norway	472454	1381	0.0029	0.000129	0.28	83.3	0.175	2.1	0.505	17.3	0.83	362.52	68590	10.52	7029.85	1209.24	1.46	5.27	3.10	0.15	0.047	0.14	0.08
Poland	4265433	101419	0.0238	0.001355	0.43	76.6	0.182	3.8	0.485	123.6	0.6	594.16	12700	6.45	695.75	282.42	0.39	1.12	4.35	0.27	0.090	0.19	0.26
Portugal	1774477	19203	0.0108	0.000943	0.10	81.1	0.221	2.8	0.473	113.0	0.66	231.23	17070	9.53	1361.26	851.83	1.51	1.97	3.33	0.27	0.096	0.17	0.21
Romania	1875887	59150	0.0315	0.001559	0.59	74.2	0.189	5.8	0.486	82.7	0.54	248.72	8830	5.74	547.68	139.58	0.47	0.89	4.03	0.16	0.077	0.11	0.30
Slovakia	820682	9939	0.0121	0.000920	0.52	76.9	0.166	5.1	0.487	112.0	0.54	104.57	15180	6.96	1030.04	269.87	0.61	1.04	4.85	0.28	0.057	0.19	0.25
Slovenia	519714	6171	0.0119	0.001479	0.42	80.6	0.202	2.1	0.498	103.7	0.55	53.59	19720	8.52	1570.97	598.61	0.73	2.30	4.13	0.25	0.075	0.19	0.27
Spain	7930528	90620	0.0114	0.000967	0.18	82.4	0.196	2.6	0.491	93.8	0.81	1281.20	22350	9.13	1926.46	809.86	1.48	2.07	2.48	0.19	0.063	0.15	0.24
Sweden	1534797	15482	0.0101	0.000754	0.26	83.2	0.200	2.1	0.501	25.2	0.88	541.06	42570	10.87	5089.96	891.74	2.04	6.09	1.90	0.18	0.042	0.15	0.07

Based on the data of the present study, CFR varies impressively within the 30 countries of the EEA, ranging from a minimum of 0.1% in Iceland to a maximum of 4.0% in Bulgaria.

Of interest, COVID-19 OMR follows a similar pattern, ranging from 59 (Iceland) to 2,333 deaths per million residents per year (Bulgaria), given that 1.978 years elapsed from January 24, 2020, when the first documented case of SARS-CoV-2 infection was detected in Europe; OMR is strongly correlated with CFR (Spearman’s rho = 0.891, P = 4x10-11).

Correlations between analyzed parameters and COVID-19 CFR

**Figure 1:**
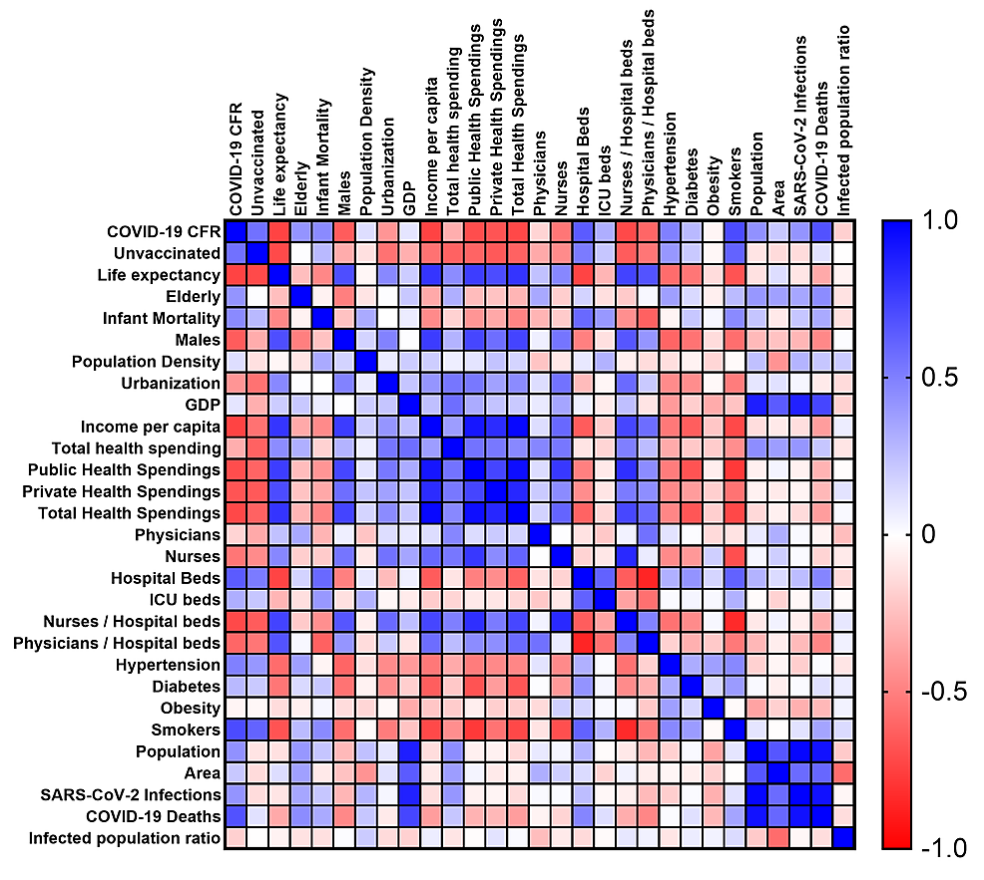
Heat map based on Spearman’s correlation coefficients including all parameters analyzed.

A detailed heat map depicting correlation coefficients between all parameters analyzed in the present study is provided in Figure 1. As COVID-19 CFR distribution deviates from normality (Kolmogorov-Smirnov P = 0.023; Shapiro-Wilk P = 0.001), the non-parametric Spearman's correlation coefficient ρ (rho) was used.

All parameters but infected population rate, population density, GDP, total health spending as % of GDP, ICU beds, diabetes, and obesity demonstrated a statistically significant correlation with COVID-19 CFR. Detailed data are presented in Table 2.

**Table 2:** Univariate analysis using Spearman's correlation coefficients between COVID-19 CFR and evaluated public health parameters; P-values have been corrected using the Benjamini-Hochberg correction. CFR: Case fatality rate.

Parameter	Spearman’s rho	P-value
Infected population (ratio)	-0.187	0.322
Unvaccinated population (ratio)	0.556	0.003
Life expectancy (years)	-0.740	3x10^-5^
Elderly population (ratio)	0.418	0.036
Gender disparity (males/total ratio)	-0.642	4x10^-4^
Infant mortality (per 1,000 live births)	0.459	0.011
Population density (residents per km^2^)	0.127	0.558
Urbanization (ratio)	-0.416	0.036
GDP (millions $)	0.101	0.626
Income per capita ($)	-0.738	3x10^-5^
Total health spending (% of GDP)	-0.320	0.117
Public health spending per capita ($)	-0.698	2x10^-5^
Private health spending per capita ($)	-0.673	2x10^-4^
Total health spending per capita ($)	-0.719	5x10^-5^
Hospital beds (per 1,000 residents)	0.517	4x10^-4^
ICU beds (per 100,000 residents)	0.316	0.117
Physicians (per 1,000 residents)	-0.170	0.432
Nurses (per 1,000 residents)	-0.539	0.004
Physicians/Hospital beds (ratio)	-0.601	4x10^-4^
Nurses/Hospital beds (ratio)	-0.715	9x10^-6^
Physicians/ICU beds (ratio)	-0.363	0.048
Nurses/ICU beds (ratio)	-0.484	0.007
Hypertension (ratio)	0.485	0.013
Diabetes (ratio)	0.274	0.179
Obesity (ratio)	-0.030	0.876
Smoking (ratio)	0.705	7x10^-5^

**Figure 2:**
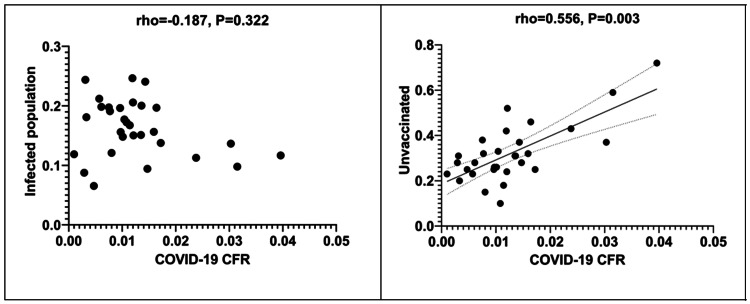
Scattergrams depicting correlations between infected population rate as well as unvaccinated population rate and COVID-19 CFR. CFR: Case fatality rate.

Scattergrams depicting correlations between the infected population rate and the unvaccinated population rate and COVID-19 CFR are shown in Figure 2.

**Figure 3:**
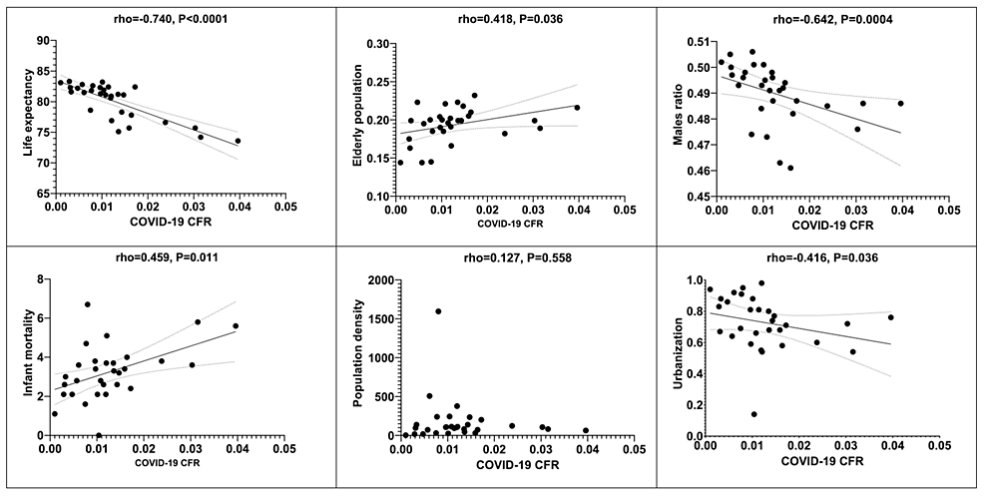
Scattergrams depicting correlations between demographics and COVID-19 CFR. CFR: Case fatality rate.

Similarly, scattergrams depicting correlations between demographics (life expectancy, elderly population rate, males ratio, infant mortality, population density, and urban population ratio) and COVID-19 CFR are shown in Figure 3.

**Figure 4:**
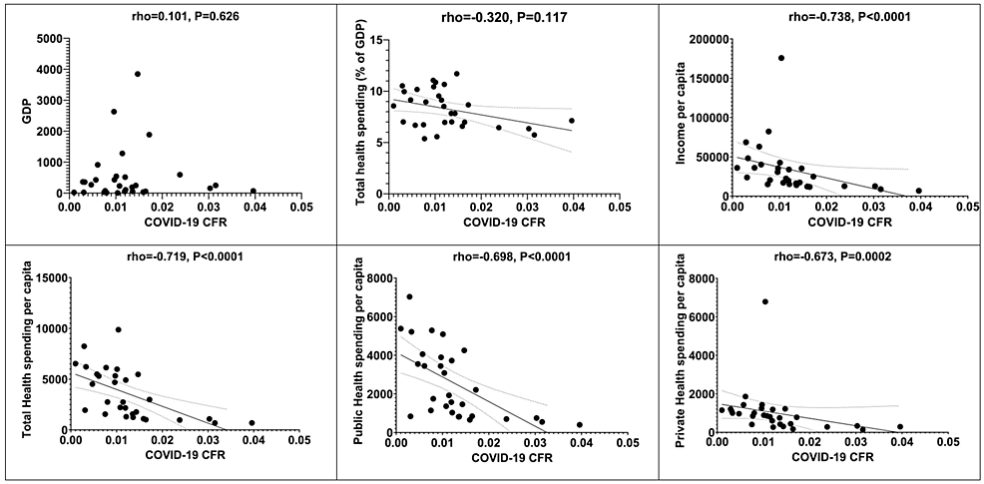
Scattergrams depicting correlations between financial indices and COVID-19 CFR. CFR: Case fatality rate.

Moreover, scattergrams depicting correlations between financial indices (GDP, total health spending as % of GDP, income per capita, total health spending per capita, public health spending per capita, public health spending per capita, and private health spending per capita) and COVID-19 CFR are depicted in Figure 4.

**Figure 5:**
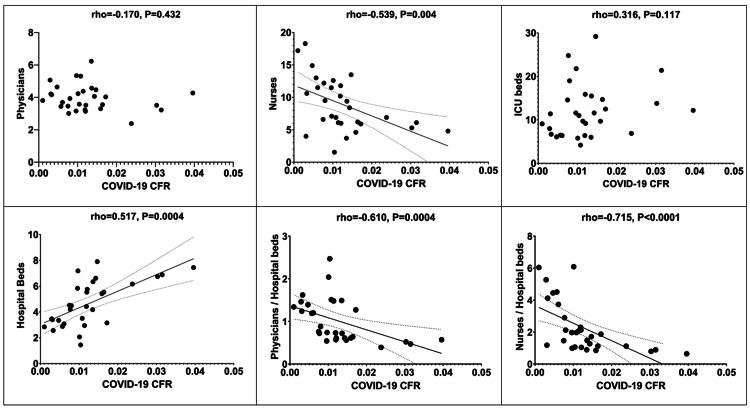
Scattergrams depicting correlations between personnel and infrastructure indices and COVID-19 CFR. CFR: Case fatality rate.

Concerning the potential relationship between personnel as well as infrastructure indices (physicians per 1,000 residents, nurses per 1,000 residents, ICU beds per 100,000 residents, hospital beds per 1,000 residents, physicians/hospital beds ratio, and nurses/hospital beds ratio) and COVID-19 CFR, the relevant scattergrams are depicted in Figure 5.

**Figure 6:**
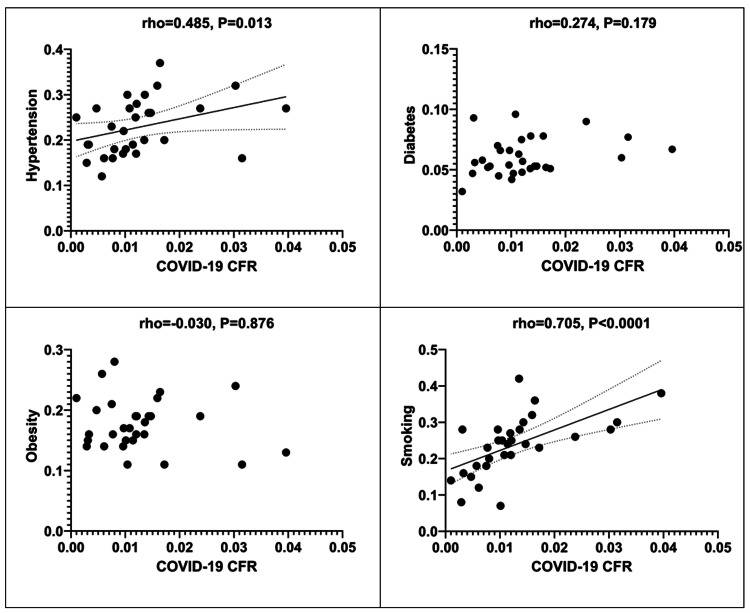
Scattergrams depicting correlations between comorbidity indices and COVID-19 CFR. CFR: Case fatality rate.

Lastly, the relevant scattergrams regarding the correlations between the examined comorbidity indices (hypertension, diabetes, obesity, and smoking) and COVID-19 CFR are provided in Figure 6.

An attempt to construct the most parsimonious multivariate model revealed that seven variables including elderly rate, male ratio, nurses/hospital beds ratio, physicians/hospital beds ratio, public health spending, smoking, and unvaccinated population rate were demonstrated to present an independent correlation with COVID-19 CFR (Table 3).

**Table 3:** Multivariate analysis using optimal scaling and ridge regression; regularization R^2 = 0.667, overall ANOVA P = 0.006.

	Cut-off after discretization	Standardized coefficient	SE†	F	Tolerance	P-value
Elderly population ratio (>65 years old)	<0.20 vs ≥0.20	0.105	0.050	4.332	0.848	0.018
Male ratio	<0.493 vs ≥0.493	-0.129	0.047	7.433	0.450	0.013
Nurses/Hospital beds	<2.30 vs ≥2.30	-0.127	0.033	15.137	0.206	0.001
Physicians/Hospital beds	<0.88 vs ≥0.88	-0.098	0.041	5.809	0.566	0.026
Public health spending	<2000 vs ≥2000	-0.113	0.041	7.525	0.270	0.013
Smokers ratio	<0.22 vs ≥0.22	0.113	0.042	7.423	0.252	0.013
Unvaccinated population ratio	<0.34 vs ≥0.34	0.219	0.052	17.470	0.702	5x10^-5^

**Figure 7:**
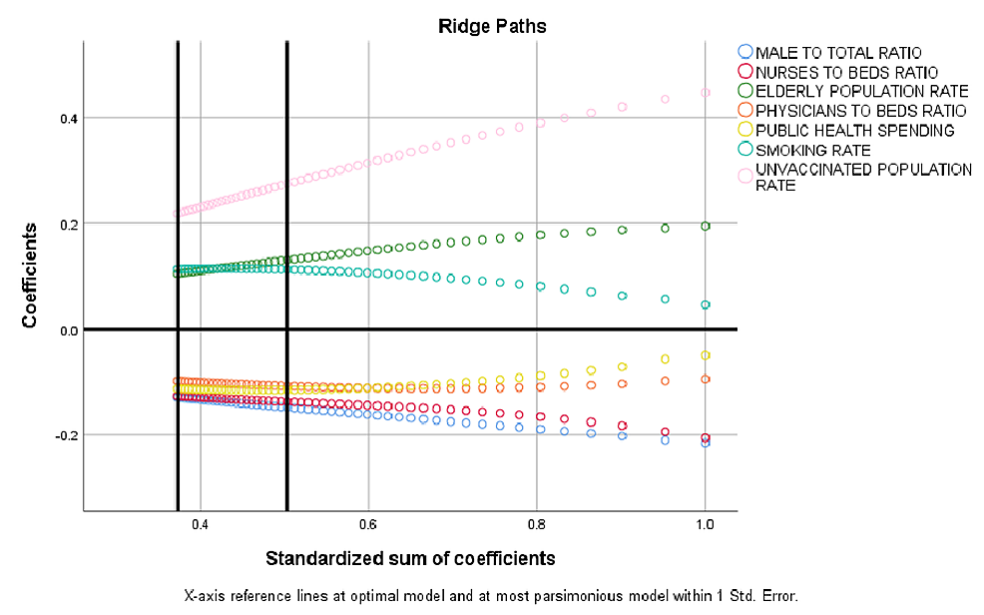
Ridge paths of the most parsimonious model; seven variables were demonstrated to present independent correlation with COVID-19 CFR. CFR: Case fatality rate.

The model was based on optimal scaling, discretization of independent variables into a maximum of seven categories, ridge regression regularization, and 10x cross-validation incorporating COVID-19 CFR as dependent variable (continuous) and all other parameters of interest as independent ones, explaining 66.7% of the variability (regularization R2 = 0.667) (Figure 7).

Low population vaccination rate is independently correlated with increased COVID-19 CFR

Unvaccinated population rate is correlated with increased COVID-19 CFR (Table 2). The most parsimonious model documented that vaccination rate revealed the strongest independent correlation with COVID-19 CFR when compared to the other six parameters included in the model. In detail, the present study has concluded that country-specific COVID-19-related CFR is estimated to increase by e0.219=1.24 times in case of vaccination rate <0.34 while holding all other independent variables of the model constant (Table 3). Controversially, the IFR has not been correlated with the CFR of the disease; this was not unexpected, as the IFR represents only a measure of the adequacy of testing and the virus spread [60].

Demographics and COVID-19 CFR: Elderly are vulnerable, whereas men might be the weaker sex

The elderly population (>65 years old) rate is linked with high CFR from COVID-19 (Table 2). This finding is consolidated as an elderly population rate ≥0.20 has been demonstrated to independently correlate with increased CFR from COVID-19 in multivariate analysis. Of note, the country-specific COVID-19 CFR is estimated to increase by e0.105=1.11 times in the case of elderly population rate ≥0.20 while holding all other independent variables of the model constant (Table 3).

The larger the men/total ratio, the higher CFR from COVID-19 was observed (Table 2). The male/total ratio has been shown to be independently correlated with increased country-specific CFR of COVID-19; an increase by e0.129=1.14 times is awaited in case of values ≥0.493 while holding all other independent variables of the model constant (Table 3).

Other demographics of interest such as life expectancy, infant mortality, and urbanization, though revealed a statistically significant correlation with COVID-19 CFR in univariate analysis (Table 2), failed to document its independent and potentially etiological nature (Table 3).

The more we invest in public health, the more lives we save: Fewer public health spending per capita is correlated with inflated COVID-19 CFR

The increased income per capita and total health spending per capita (either public or private) are correlated with lower COVID-19 CFR (Table 2). In multivariate analysis, the public health spending per capita, and not the private one, has been recognized as the most significant financial public health parameter to interfere with the disease. In that case, the COVID-19 CFR is estimated to increase by e0.113=1.12 times in the case of countries spending <2,000$ annually per capita for public health while holding all other independent variables of the model constant (Table 3). Interestingly, GDP is not linked with COVID-19 CFR, neither itself nor as % attributed to total health spending.

Country health profiles and COVID-19 CFR: a lesson to prioritize personnel rather than infrastructure

Both physicians and nurses, when considered as ratios to hospital beds or ICU beds, revealed a strong, negative relationship with COVID-19 CFR, despite that in absolute numbers, the relevant correlations are weaker (in case of nurses), or absent (in the case of physicians). Of note, ICU beds were not correlated with the outcome of interest, and hospital beds presented a positive correlation with CFR of COVID-19 (Table 2). When the multivariate analysis was performed, the number of nurses and physicians per hospital bed remained independently correlated with the CFR of COVID-19. According to the present model, COVID-19 CFR is estimated to increase by e0.127=1.14 and e0.098=1.10 times in the case of countries with <2.30 nurses and <0.88 physicians per hospital bed, respectively, while holding all other independent variables of the model constant (Table 3).

Smoking reserves a key role among comorbidities affecting COVID-19 CFR

Hypertension and smoking are the two most prominent public health issues regarding COVID-19 CFR (Table 2). Smoking retained an independent correlation with increased CFR of the disease in the multivariate analysis model. In countries with a smoking ratio ≥0.22, the COVID-19 CFR is awaited to increase by e0.113=1.12 while holding all other independent variables of the model constant (Table 3). Diabetes and obesity, though documented to be comorbidities affecting outcome after SARS-CoV-2 infection, failed to present a statistically significant correlation with COVID-19 CFR in univariate analysis (Table 2).

## Discussion

To the best of our knowledge, this is the first study to describe the impact of SARS-CoV-2 vaccination coverage and several key public health indicators in country-specific COVID-19 CFR within the EEA. As a result, COVID-19 CFR varies substantially among EEA countries, and is independently linked with low vaccination rate (<0.34), elderly population rate (≥0.20), limited public health spending (<2,000 $ per capita), insufficient physicians per hospital beds ratio (<0.88), inadequate nursing personnel per hospital beds ratio (<2.30), and high prevalence of smoking habits (≥0.20). Vaccination coverage, public health financing, hospital staffing with both physicians and nursing personnel, and anti-smoking campaign planning are thus believed to be critical to prevent increased COVID-19 CFR.

It has been recognized that vaccination status is linked with lower death rates. In detail, even a single dose of mRNA BNT162b2 vaccine has been reported to reduce the risk of death significantly (OR: 0.51, 95% CI: 0.37-0.62). Moreover, the vaccine prevented 96.7% of COVID-19-related deaths in vaccinated individuals [61,62]. Both ChAdOx1-S and BNT162b2 vaccines retain 84.8% (95% CI: 76.2 to 90.3) and 91.9% (95% CI: 88.5 to 94.3) effectiveness in reducing the risk of death, respectively [63]. Abdul Taib NA et al. reported that the mortality rate among vaccinated people was consistently lower than those who were unvaccinated. Moreover, the mortality rate of those who received inactivated vaccines was higher than the recipients of the BNT162b2 and ChAdOx1 vaccines [64]. Countries such as Switzerland, USA, Chile, and England, being all outside EEA, offer paradigms of significantly higher death rates among the unvaccinated population, when compared with those who had been fully vaccinated against SARS-CoV-2, especially after booster dose [65]. Correspondingly, the present study has concluded that COVID-19-related CFR is estimated to increase by 24% in countries with vaccination rates <0.34.

According to a recently published study, almost half (~47%) of the variation observed in country-specific COVID-19 CFR is attributed to the country's age profile. In comparison, another 44% could not be explained [66]. An analysis of age-specific data from 20 countries from Europe and Northern America concluded that >80% of the variance of country-specific COVID-19 CFR could be attributed to the proportion of the elderly population (>75 years of age). However, the authors did not investigate the role of other parameters contributing to mortality from COVID-19 [67]. Abdul Taib NA et al. and Soneji S et al. concluded similar results [64,68]. Interestingly, García CN claimed that not the elderly population rate, but rather the institutionalization of elderly people in long-term care resources (as care homes) might explain the statistically significant contribution of aging to COVID-19 mortality [69]. Concerning gender disparity, male sex is linked with increased COVID-19 CFR worldwide [70]. This observation has been supported to be independent of age or comorbidities [71]. The issue of standardization not only to age but also to sex is further underlined, focusing primarily on the effect of the decreasing life expectancy in age and sex impact on taming COVID-19 mortality [72]. The present study has demonstrated that the country-specific COVID-19 CFR is estimated to increase by 11% and 14% in countries with elderly population rates ≥0.20 and male/total ratio ≥0.493, respectively.

The role of public health financing and access to health care in COVID-19 CFR has not been broadly investigated. Mackey K et al. report that African American/Black and Hispanic populations had experienced disproportionately higher rates of SARS-CoV-2 infection and COVID-19-related mortality but similar CFR [73]. Interestingly, strong positive associations of Black, American Indian, and Alaska Native versus White race and urban versus rural residence with SARS-CoV-2 CFR were observed, implying the existence of racial and geographic barriers concerning the accessibility of health care facilities [74]. However, whether differences in health care access and exposure risk may be driving higher infection and mortality rates yet remains unclear. Of interest, the present study underlined the major and independent positive impact of public health spending in attenuating COVID-19 CFR, estimating an increase by 12% in the case of countries spending <2,000$ annually per capita for public health.

A significant non-linear relationship between COVID-19 CFR and the number of physicians (P ≤0.001), and nurses and midwives (P ≤0.001) had been documented [75]. This finding is also in keeping with the present study results, which underlined that COVID-19 CFR is estimated to increase by 14% and 10% in the case of countries with <2.30 nurses and <0.88 physicians per hospital bed, respectively. The latter implies that adequacy is urged not only to upgrade the infrastructure level (hospital beds and ICU beds) but also to staff it with the required qualified nursing and medical personnel. Taken together with the already demonstrated beneficial impact that the increased public health spending has on taming COVID-19 CFR, it is reasonable to support that the available public health investments have to be prioritized towards recruiting additional nurses and physicians.

COVID-19 mortality rates were also associated with comorbidities, as described in a recently published study. However, the risk of death by specific comorbidity type has not been conclusively assessed [65]. Hypertension and diabetes have been reported to increase COVID-19 CFR; despite that the elderly had a higher CFR, the relevant risk ratios were more pronounced among the younger population [76]. Moreover, obesity has been demonstrated to be an independent risk and prognostic factor for the severity of the disease [77]. These observations seem to disagree with the findings of the present study, which reveal comparable country-specific CFR after adjustment for age and sex. However, detailed data concerning undiagnosed or uncontrolled hypertensive or diabetic patients that could further elucidate this issue are not available. On the other hand, smoking had been early recognized as a risk factor for dying from COVID-19, too. Compared to former and never smokers, current smokers were at greater risk of severe complications and higher mortality rate [78]. This is in keeping with the independent correlation between the smoking ratio and country-specific COVID-19 CFR observed by our study. In contrast, the increase of COVID-19 CFR attributable to smoking has been estimated to be 12% in countries presenting smokers ratio ≥0.22. Other factors that have not been included in the present study, such as air travelers (who might reflect mobility) or isolated territories (as isles), have been demonstrated to present a positive impact on mortality from COVID-19 [63]. Of interest, the tuberculosis rate was proposed to be associated with COVID-19 CFR [79]. A more complex model introduced population density, population density/GDP, urbanization ratio/GDP, hospital beds/GDP, the gender ratio among elderly (>80 years), population density/tuberculosis incidence, average temperature, and average temperature/GDP as prognosticators of logarithmic COVID-19 mortality [80].

OMR could be argued to provide a safer estimate of the risk of dying from COVID-19 when compared to CFR, considering that COVID-19-associated deaths are rigorously registered and the relevant population at risk might be precisely approached, as merely everyone had been eventually exposed to SARS-CoV-2. However, OMR is suitable neither as a measure of the disease's severity nor its lethality but rather as an estimate of individual risk of dying from the disease. Moreover, OMR depends on total population and time; the latter parameter is crucial considering that the period elapsed from SARS-CoV-2 infection to outcome, though generally short, may vary substantially. Under these circumstances, CFR and OMR could not be interchangeably used in practice, at least in the case of COVID-19 disease.

The major limitation of the present study is the inability to exhaustively examine all factors that potentially contribute to COVID-19 CFR. The main reason for this compromise is that too many variables usually produce multivariate models with over-fitting or collinearity issues. Especially, data regarding the specific SARS-CoV-2 variants or the time of seeking medical help from the onset of the disease are lacking, thus blurring the results as far as the potential contribution of these parameters to CFR is concerned. Additionally, it could be argued that a time-dependent analysis could offer a more realistic approach, given that COVID-19 CFR has been declining since May 2020 [81]. Moreover, the impact of psychological profile, social attitude, and compliance with governmental planning has not been taken into account, as precise measurements are unfeasible.

## Conclusions

In conclusion, COVID-19 CFR, which varies substantially among EEA countries, is independently correlated with low vaccination rate, increased elderly population rate, diminished public health spending per capita, insufficient physicians and nursing personnel per hospital beds, and prevalent smoking habits. Therefore, European and national public health authorities could consider these parameters when prioritizing actions for the management of the SARS-CoV-2 pandemic.
